# Development and validation of the geriatrics health behavior questionnaire (GHBQ)

**DOI:** 10.1186/s12889-022-12927-1

**Published:** 2022-03-17

**Authors:** Maryam Bakhshandeh Bavarsad, Mahshid Foroughan, Nasibeh Zanjari, Gholamreza Ghaedamini Harouni, Zahra Jorjoran Shushtari

**Affiliations:** 1 Iranian Research Center on Aging, Department of Aging, University of Social Welfare and Rehabilitation Sciences, Tehran, Iran; 2 Iranian Research Center on Aging, University of Social Welfare and Rehabilitation Sciences, Tehran, Iran; 3 Social Welfare Management Research Center, University of Social Welfare and Rehabilitation Sciences, Tehran, Iran; 4 Ph.D., Social Determinants of Health Research Center, University of Social Welfare and Rehabilitation Sciences, Tehran, Iran

**Keywords:** Older adult, Geriatrics, Psychometrics, Reliability, Validity, Health behavior

## Abstract

**Background:**

Considering the importance of health behaviors in health outcomes, it is necessary to assess health behaviors precisely. This study aimed to develop and validate The Geriatrics Health Behavior Questionnaire among Iranian older adults.

**Methods:**

This cross-sectional and methodological study was conducted on 420 community older adults (age ≥ 60) through random multi-stage sampling. The initial questionnaire has been developed with 22 items and seven subscales based on an extensive literature review, evaluation of related questionnaires, and experts’ opinions. Face and content validity were evaluated by interviewing 10 older adults and 18 specialists. The construct validity was evaluated via Known-groups validity and convergent validity. The reliability of the questionnaire was calculated by internal consistency, test-retest, and absolute reliability.

**Results:**

The face validity was conducted by using interviews with older adults and gathering the specialists’ opinions. The items were grammatically and lexically corrected accordingly. Two items were deleted due to CVR < 0.44. Modified Kappa statistic (K*) and I-CVI for all items were higher than 0.88. The average content validity index (S-CVI/Ave) value was 0.94. Three items were deleted to improve the internal consistency; the final GHBQ consisted of 17 items with Cronbach α = 0.72. Acceptable convergent validity was approved by a significant correlation between GHBQ and SF8™ health survey (*r* = 0.613, *P* value< 0.001). Independent t-test showed that older adults with education level ≥ high school have significantly higher health behavior scores than those with education level < high school (11.93 ± 2.27 vs. 9.87 ± 2.35, t = − 9.08, *p* < 0.001). Intra-class correlation coefficient (ICC) for the total questionnaire was 0.92 (95% CI =0.84 to 0.96). Standard Error Measurement (SEM) and Minimal Detectable Change (MDC_95_) were 0.71 and 1.98, respectively.

**Conclusion:**

The present study results showed that the Geriatrics Health Behavior Questionnaire had suitable validity and reliability among Iranian older adults. It is recommended to consider its comprehensiveness and yet its briefness in other populations after passing validation.

**Supplementary Information:**

The online version contains supplementary material available at 10.1186/s12889-022-12927-1.

## Background

The population of Iran is growing old, similar to other developing centuries. The overall percentage of the Iranian older adults population is expected to increase from 5.4% in 1986 to 21.7% in 2050 [1]. One of the most important aims of healthy people 2020, developed by the US Department of Health and Human Services (2010), is to enhance healthy behavior for all ages. Such plans help grow a population of older adults who have a healthy lifestyle [2]. A healthy lifestyle is a comprehensive concept that includes behaviors such as alcohol and tobacco use, Sexual activity, sedentary behavior, exercise, diet, stress management, medical adherence, and check-ups [3–10]. Row and Kahn (1997) showed that older adults in the successful aging category actively search to find a lifestyle that promotes their quality of life and health [11]. Previous studies showed that healthy behaviors play a key role in health and vitality. A systematic review showed that a healthy lifestyle has potential benefits to protect cognitive health in later life and reduce the risk of dementia and cognitive decline [12]. Also, some pieces of evidence support the protective effects of healthy lifestyle behaviors on health outcomes such as better sleep quality, physical health [6], quality of life, the lower rate of stress, health distress, depression, frailty [13], diabetes [14], perceived hearing difficulties [15], and reduced mortality risk [7, 8, 16].

Considering the importance of health behaviors in health outcomes, a precise assessment of health behaviors is necessary. This necessity has led to the development of a wide range of instruments, some of which are general and others are age-specified. HELP–Screener is a 15-item questionnaire that has been developed for older adults. It is a valid, time-efficient, easy-to-understand questionnaire with yes/no responses. It has included various aspects of a healthy lifestyle such as exercise, diet, socialization, leisure, and spirituality; however, this questionnaire has not separated dimensions [2]. The Health Promoting Lifestyle Profile II (HPLP-II) is a general questionnaire that included 52 items in six subscales (nutrition, physical activity, health responsibility, stress management, interpersonal relationships, and spiritual growth) [17]. It is a cross-cultural questionnaire that has been translated into different languages, including Persian, Malaysian, Spanish, Brazilian, Chinese, and Turkish [18–23]. Tanjani et al. (2016) showed that HPLP-II is a suitable tool for assessing health behaviors among older adults [21], but Padula (1997) emphasized that some of the HPLP items are not appropriate for the older adults population [3]. Although HPLP-II is an appropriate tool and covers various dimensions of health behaviors, it is too long and is not user-friendly to assess older adults, especially when it is integrated with other questionnaires simultaneously. The Health-Promotion Activities of Older Adults Measure was designed by Padula in 1997 [3]. It is a 44-item instrument with five subscales: Collaborative Health Management/injury Prevention, Stress Reduction/Rest and Relaxation, Exercise, Substance Abuse Prevention, and Nutrition. This instrument’s strength is that it has been designed specifically for older adults, but it is too long as HPLP-II.

Most of the studies use several items to assess health behaviors instead of a specific questionnaire (e.g., “How often in the past month did you eat (fresh) fruit?”, ‘Have you gained more than 10 pounds in the last 6 months, ‘Do you do vigorous exercises for 15–30 min or more at least three times a week?’,…) [8, 24–28] which their validity and reliability are not clear. This study aimed to develop and validate a practical, short, and simple instrument to cover all aspects of health behaviors for assessing Health Behaviors among older adults.

## Methods

### Study design

This is a cross-sectional, methodological study that was conducted in Tehran, Iran. Tehran is the capital of Iran, with various patterns of ethnicities, subcultures, and socio-economic levels.

### Item generation

The concept and dimensions of health behaviors have been drawn out by a deductive approach. The initial questionnaire has been developed based on an extensive literature review, evaluation of related questionnaires, and experts’ opinions. Literature was searched on the different databases, including Scopus, PubMed, Web of Science, and Google scholar, with the following keywords: health behavior, lifestyle, health promotion, older adult, aging, and elderly. We did not limit the search to a specific time interval. The instruments and items used to assess health behaviors among older adults in different studies were extracted and then assessed by the research team. The following questionnaires were used for developing the items of GHBQ: HPLP- II [17], Health Promotion Activities of Older Adults Measure [3], Morisky Medication Adherence Scale (MMAS) [29]. The selected items by the research team were sent to the scholars of gerontology, geriatrics, nursing, psychology, and social sciences, and their comments were gathered, assessed, and implemented on the questionnaire. Experts’ opinions helped us to select and arrange appropriate dimensions and items based on their experience.

The initial questionnaire was generated with 7 dimensions, including physical activity (2 items), nutrition status (3 items), medication adherence (4 items), stress management (6 items), smoking and alcohol consumption (2 items), sleep quality (2 items) and check-ups (3 items).

The final Geriatrics’ health behavior questionnaire (GHBQ) includes 17 items on seven dimensions: physical activity (1 item with 2 parts), nutrition status (2 items), medication adherence (4 items), stress management (4 items), smoking, and alcohol consumption (2 items), sleep quality (2 items) and medical check-ups (2 items). GHBQ items are scored between 0 and 1 based on the accumulation approach as previously used to create the frailty index [30]. Total questionnaire scores range between 0 and 17, and higher values show better health behaviors. The final 17-items questionnaire and the scoring method are presented in Appendix 1.

### Content related validity

In this study, the content-related validity includes face validity and content validity.

#### Face validity

Both Qualitative and Quantitative face validity were evaluated in this study. We interviewed 10 older adults selected by the convenience sampling method to evaluate items’ difficulty, relevancy, and ambiguity. Then the research team assessed the interviews and all of the comments implemented to edit statements.

Impact score formula (Frequency (%) × Importance) was used to evaluate quantitative face validity. Items with an impact score ≥ 1.5 were considered appropriate for more analysis [31].

#### Content validity

The qualitative content validity was used to assess the statements according to grammar, wording, item allocation, scaling, clarity, and simplicity. Eighteen specialists consisting of gerontologists, geriatricians, geriatric nurses, psychologists with a Ph.D. degree were asked to provide feedback to edit and revise the statements.

Quantitative content analysis was applied by two indexes, namely content validity ratio (CVR) and content validity index (CVI). CVR and CVI indices assess the necessity and relevancy of the Items, respectively. In the present study, relevancy is considered as the only index to evaluate CVI, according to Polit et al.’s suggestion [31, 32].

To calculate the CVR index, first, each item was rated by experts according to a 3-point scale from not necessary to essential. Then the CVR index was estimated by using this formula: CVR = (nE − N/2)/(N/2), where nE signifies the number of panelists indicating “essential” and N is the total number of panelists. The accepted CVR value, based on the critical value of Lawshe’s table and the number of subject matter experts (18 experts), was considered > 0.444 [33].

Content validity index was assessed for each item, which was answered on a 4-point scale from not relevant to completely relevant. I-CVI was calculated by dividing the number of experts giving a rating of ‘3’ or ‘4’ to each statement by the total number of experts. An I-CVI score over 0.79 was considered adequate. Scale-CVI was calculated based on S-CVI/Avg, where the sum of I-CVI was divided by their numbers. Based on Polit’s opinion, acceptable S-CVI/Avg is equal to or more than 0.9.

The study used modified kappa statistics to evaluate the chance agreement between several raters. So the present study used the modified Kappa statistic (K*), which was designed by Polit et al. K* > 0.74 is considered excellent [31]. The probability of chance agreement was first calculated using the following formula: pc = [N ! /A ! (N − A)!] ∗ 〖0.5〗 ^ N, where N signifies the number of experts and A is the number of experts who agreed that the item was relevant. Then having calculated I-CVI for all items, kappa was computed by the following formula: K ∗  = (I − CVI − pc)/(1 − pc).

### Sampling

This study selected 420 eligible community-dwelling older adults (age ≥ 60 years, with adequate cognitive functioning and ability to communicate) by multi-stage cluster sampling. At first, 22 districts of Tehran were classified into five groups in terms of socio-economic development levels, from developed areas to underdeveloped (very poor) areas [34]. One district in each cluster and then two regions in each district were selected randomly. The sample size in each district was determined based on its population ratio to the overall population. In terms of Intra-class correlation coefficient (ICC), 30 older adults were selected to complete the questionnaire twice, 2 weeks apart. All questionnaires have been completed by using door-to-door surveys.

### Construct validity

The Geriatrics’ Health Behavior Questionnaire construct validity was evaluated via Known-group validity and convergent validity**.**

#### Convergent validity

Convergent validity was assessed via the associations with other measures, including health-related quality of life (SF8™), related to health behaviors. Most of the studies report a significant relationship between health behaviors and quality of life; also, several studies showed the effect of health behavior interventions on quality of life [13, 35–40]. So, the present study used the SF8™ health survey [41] to evaluate convergent validity with the new scale. The SF8™, an abbreviated version of the SF 36 health survey, consists of two dimensions: physical component summary (PCS) and mental component summary (MCS) [42]. Both components have scores between 0 and 100, with higher scores showing better quality of life. Results of the SF-8™ from older adults in Iran reported Cronbach’s alpha of 0.84 and 0.79 for PCS & MCS subscales, respectively [43].

The Pearson correlation coefficient test was used to evaluate the relationship between SF8™ health survey and our Geriatric Health Behavior Questionnaire.

#### Known-group validity **(**discriminative validity)

The relation between education and health behaviors is explained by three behavioral effects. The first effect is that a person with a higher level of education can utilize resources more efficiently in order to gain healthier productions. The second one regards subjective variables such as time preference. Being aware of future effects of activities is the third behavior. This means that well-educated people live more healthily. For example, knowing the consequences of smoking will cause them to avoid this unhealthy behavior [44].

Healthier lifestyles are chosen more often by educated people based on their knowledge regarding the relationship between health behaviors and health outcomes [45]. Older adults in this study were divided into two groups based on education level (<high school, ≥high school). Then health behaviors were compared between groups using an independent t-test.

### Reliability

Internal consistency was assessed by Cronbach’s alpha, Kuder- Richardson for subscales with dichotomous choices [46], and Spearman-Brown coefficient for two-item subscales based on Eisinga et al. results [47]. Internal consistency for the total GHBQ was assessed by Cronbach’s alpha (values above 0.7 were considered acceptable) [48] and McDonald omega coefficients [49]. Kirk and Miller (1986) stated that the reliability of a questionnaire could be determined by measuring the value of the correlation between the scores of each item and the total score (item-total correlation) [50], and the value more than 0.25 is considered high based on Nunally and Bernstein (1994) study [46]. Also Cohen (1988) classified the values into three categories, small (0.10 to 0.29), medium (0.30 to 0.49) and high (0.50 to 1.00) [51]. The Pearson correlation coefficient test was used to evaluate the correlation value of each subscale Score with Total Score.

Intra-class correlation coefficient (ICC) with the two-way mixed-effects model and absolute agreement (95% confidence level) was calculated to evaluate test-retest reliability at scale level. ICC values above 0.75 were considered acceptable [52]. Also, test-retest reliability at the item level (Cohen’s (1960) Kappa coefficient) was calculated for binary items [53]. Landis and Koch guidelines (1977) were used to interpret the results. The values from 0.0 to 0.2 indicate slight agreement, 0.21 to 0.40 indicate fair agreement, 0.41 to 0.60 indicate moderate agreement, 0.61 to 0.80 indicate substantial agreement, and 0.81 to 1.0 indicate almost perfect or perfect agreement [54].

Standard Error Measurement (SEM) and Minimal Detectable Change (MDC_95_) were used to evaluate absolute reliability by the following formula: $SEM=\mathrm{SD}\sqrt{1- ICC}; MDC= SEM\times \sqrt{2}\times 1.96.$

Minimal detectable change is the minimum amount of change that must be observed to be considered a real change. Thus, a change in scores smaller than MDC^95^ can be related to measurement error [55].

### Ceiling and floor effects

Ceiling and floor effects show the content validity is not appropriate and occur when more than 15% of participants choose responses at the higher and the lower end of the scale, respectively. The ceiling and floor effects were calculated as a percentage for all data [56].

The following descriptive and analytical indices were used in the present study by SPSS 22 software; Cronbach’s alpha, Kuder- Richardson, Spearman-Brown coefficient, Pearson correlation coefficient test, intra-class correlation coefficient, independent t-test.

## Result

A total of 420 older adults consisting of 224 men (53.3%) were recruited in this study. Participants’ age ranged from 60 to 93 years, with a mean of 69.03 ± 7.61 years. Other demographic details of the participants are shown in Table 1.

**Table 1:** Demographic Characteristics of participants(*n* = 420)

Variables		n	Percent
Gender	Male	224	53.3
	Female	196	46.7
Education	<High school	210	50
	≥High school	210	50
Marriage status	Single	9	2.1
	Married	330	78.6
	Widowed	70	16.7
	Divorced	11	2.6
Socio-economic Status	Developed	124	29.5
	Moderate developed	58	13.8
	Relatively developed	115	27.4
	less developed	62	14.8
	Underdeveloped	61	14.5

### Content related validity

#### Stage 1: face validity

A total of 10 older adults between the ages of 60–76 were interviewed. Roughly 30% of participants were illiterate, 40 and 30% had diplomas and bachelor’s degrees, respectively. Fifty percent of the samples were female, and all of them were married. The impact score of all items was higher than 1.5, so no item was removed in this phase. Some items were reviewed based on older adults’ comments (e.g., physical activity was replaced by exercise and “when I am stressed, I often try to relax with listening to music, talking to someone, gardening or…” were replaced by “When I am stressed, I often try to do something such as listening to music, talking to someone, gardening or….”).

#### Stage 2: content validity

We assessed both qualitative and quantitative content validity. The result of CVR showed that two items had a value < 0.44, so both were deleted. Finally, 20 items remained to assess the content validity index. Modified Kappa statistic (K*) and I-CVI for all of the items were higher than 0.88. The average content validity index (S-CVI/Ave) value was 0.94, which showed that the total questionnaire had appropriate content validity. Also, we revised 9 items based on grammar and wording according to the experts’ panel suggestion.

### Construct validity

#### Convergent validity

Convergent validity was evident based on a significant correlation between GHBQ and SF8™ health survey (*r* = 0.613, *p* < 0.001). Also, the Pearson correlation coefficient test showed that there was a significant correlation between GHBQ and physical component (*r* = 0.598, *p* value< 0.001) and mental component of SF8™ (*r* = 0.510, *p* value< 0.001) respectively.

#### Known-group validity **(**discriminative validity)

Independent t-test showed that older adults with education level ≥ high school have more healthy behaviors than those with education level < high school (11.93 ± 2.27 vs. 9.87 ± 2.35, t = − 9.08, *p* < 0.001). The results showed significant differences between the two groups in all of the subscales except smoking and alcohol consumption (Table 2).

**Table 2:** Health behaviors comparison between groups

Subscales	<high school (***n*** = 210)		≥high school (***n*** = 210)		F	df	t	***P***-value
	Mean	SD	Mean	SD				
Check Ups	0.76	0.71	1.08	0.86	18.29	418	−4.129	< 0.001
Physical Activity	0.13	0.27	0.40	0.39	61.61	418	−7.967	< 0.001
Sleep quality	1.01	0.61	1.37	0.49	52.77	418	−6.570	< 0.001
Stress management	2.98	0.79	3.23	0.61	11.78	418	−3.543	< 0.001
Nutrition status	0.61	0.53	0.90	0.55	0.07	418	−5.325	< 0.001
Smoking and alcohol consumption	1.41	0.61	1.33	0.69	11.18	418	1.263	0.207
Medication adherence	2.93	1.27	3.60	0.93	25.84	418	−6.078	< 0.001

### Reliability

In this step, three items (one item of nutrition status and two items of stress management) were deleted to improve the internal consistency; the final GHBQ consisted of 17 items with seven subscales. The internal consistency of the total questionnaire was 0.72, and McDonald’s Omega was 0.714. The values were evaluated for each subscale presented in Table 3 that showed moderate to good internal consistency. Cronbach α has not been assessed for the physical activity subscale because of having only one item.

**Table 3:** The values of correlation and Internal consistency and ICC of subscales

Variables	Number of items	Internal consistency method	Values	ICC(95%CI)	Correlation Value of subscales Score with Total Score
Nutrition status	2	Spearman-Brown coefficient	0.57	0.80(0.60–0.90)	*r* = 0.46
Medication adherence	4	Kuder- Richardson	0.81	0.85(0.70–0.93)	*r* = 0.77
Stress management	4	Cronbach’s alpha	0.59	0.92(0.83–0.96)	r- = 0.54
Smoking and alcohol consumption	2	Spearman-Brown coefficient	0.62	1	*r* = 0.18
Check-ups	2	Kuder- Richardson	0.53	0.86(0.71–0.93)	*r* = 0.39
Sleep quality	2	Spearman-Brown coefficient	0.53	0.98 (95% CI =0.97–0.99)	*r* = 0.60

Pearson Correlation coefficient test showed a medium to high correlation value of the subscales scores with the total score of questionnaire except for the subscale of Smoking and alcohol consumption that the value was small. Correlation values of physical activity and sleep quality subscales with the total score were (*r* = 0.47 and 0.60 *p* < 0.001), respectively (Table 3). Item-total (biserial) correlations showed that there is a significant correlation between each item and the total score. The biserial correlation values were between 0.13 and 0.69 (*p* < 0.01), where the minimum value belongs to the item about alcohol consumption, and the maximum belongs to medication adherence. Based on Cohen’s (1988) classification [51], six items had a high correlation, 6 had a medium correlation, and 5 had a small correlation with the total score. The test-retest method and the Intraclass correlation coefficient (ICC) were used to calculate the stability of the questionnaire. ICC for the total questionnaire was 0.92 (95% CI =0.84 to 0.96) ICC for the physical activity was 0.98 (95% CI =0.97–0.99) (Table 3). Kappa coefficients for binary items, including Medication adherence and check-ups, were 0.43 to 1, indicating moderate to perfect agreement.

Standard Error Measurement (SEM) and Minimal Detectable Change (MDC_95_) were 0.71 and 1.98, respectively. The ceiling and floor effects for the total questionnaire were 0%.

## Discussion

Various tools have been designed to assess healthy lifestyles and health behaviors [2, 3, 17]. Some of these questionnaires have also been validated in the older-adults population [21]. Although psychometric assessment of questionnaires is common in various age groups, due to the specific characteristics of older adults, it is recommended to design questionnaires with simple, short, and clear items for this age group. The number of items is one element that requires consideration when designing questionnaires for older adults. Too many items put a burden on the respondents. Older adults usually become tired of answering long items sooner than other age groups. A majority of studies about older adults’ health behaviors have replaced questionnaires with a collection of questions [8, 57–60]. However, the main limitation of these questions is the vagueness of their validity and reliability. The current study has tried to develop a short questionnaire suitable for the senior community, considering objective questions and covering different dimensions. The current questionnaire includes physical activity, nutrition status, medication adherence, sleep quality, stress management, check-ups, smoking, and alcohol consumption. This study is the first step out of the lengthy process of psychometric validation, and it primarily focuses on content and construct validity.

Generally, Questionnaires designed to study health behaviors ignore medication adherence, while this dimension is of great importance in the older-adults population. Medication adherence is more important among older adults than other age groups due to multiple chronic diseases from which they usually suffer [61]. Therefore, it should be considered in health behaviors. In the current questionnaire, one dimension has been dedicated to this concept. Most studies point to breast examination, Pap smear, and prostate test for screening [25, 62, 63]. However, based on the United States Preventive Services Taskforce (USPSTF) guidelines, the PSA test, self-breast examination, and Pap smear during older ages are considered in grade D and not recommended for older adults [64]. It appears that having questions about these behaviors is not suitable for evaluating health behaviors. Thus, general annual physical exams and dental check-ups have been used in the current study.

Another dimension focused on in this questionnaire was sleep quality. Sleep is important at all ages; however, it is highly crucial in older ages due to older adults’ problems, such as restless leg syndrome, taking various medicines, and environmental changes [65]. Sleep disorder is recognized as a geriatrics syndrome [64]. Complications of poor sleep can cause irreparable harm to older adults’ health, such as fractures [66]. Two items in the present questionnaire investigated both the quality and quantity of sleep in older adults. Sleep duration of 7–8 h [8, 57] or 7–9 h [55, 56] is considered normal for older adults. National sleep foundation advises between 7 and 9 h of sleep per night for adults aged 26–64 and 7–8 h of sleep for 65 years and older [67]. Since the current study has been conducted in a developing country, old age was considered over 60. As a result, the optimal sleep duration was decided to be 7–9 h. In the case of using this questionnaire for older adults of 65 years old and above, it is recommended that the range of 7–8 h is considered the optimal sleep duration.

Based on health department guidelines, physical activity of 150 min or more per week is recommended for older adults [68]. Hence, in the present questionnaire, this objective criterion was used to assess older adults’ physical activity by simply asking two questions (Appendix 1).

Regarding healthy nutrition, there are several factors, such as proper consumption of salt, oil, fish, and vegetables. According to the World Health Organization and Food and Agriculture Organization, a minimum of five portions of fruit and vegetables per day is suggested [69]. Most studies consider daily consumption of fruit and vegetables as a suitable criterion for a healthy lifestyle [57, 58, 60, 70, 71]. In this questionnaire, examples have been given to older adults to facilitate understanding fruit and vegetable portions.

When designing a questionnaire, both stems of the questions and response options are critical. A large variety of choices leads to better data collection; however, it may be exhausting or confusing to older adults. Previous experience shows that older adults resisted answering multiple-choice questions. This may be due to their difficulty in information retrieval and unfamiliarity with such questions [72]. In the current questionnaire, the response options, tailored to each item, are designed to cause the least fatigue and complexity for older adults and minimize response time. For example, short-answer questions have been used regarding physical activity and sleep. Also, two-choice yes/no questions have been used regarding medication adherence and screening.

Various methods were used to estimate the validity of the designed scales. For instance, face validity was used to simplify the sentences and make them understandable; the sentences were edited based on the comments from older adults and experts. The modified Kappa statistic and content validity index results indicated that the Geriatrics Health Behavior Questionnaire had a desirable content validity. Subscales score had a moderate to high correlation with the total score, indicating proper validity of the questionnaire. Known-group and convergent validity were also used to estimate the construct validity. The results proved that the current questionnaire not only had a good correlation with health and its dimensions (mental and physical health), but it was also able to differentiate between groups (educated versus uneducated). Although the results of our study showed no significant difference between educated and uneducated individuals in terms of alcohol consumption and smoking, it can be seen that there are similar cases in previous studies, such as in the study of Pärna K et al. (2014) [73]. In addition, different results were reported in various cohorts so that there was no relationship between education and smoking in the population of men in 1990–1994 and women in 1990–2000 [73].

In the present study, all three types of reliability were examined: the current questionnaire has very desirable reliability. The rate of absolute reliability based on standard error measurement was calculated below 10% of the total score (0.71), indicating desirable reliability. Moreover, the minimum detectable change was determined 1.98 for this questionnaire, indicating that equal or greater changes with a 95% confidence level are not due to measurement error or differences in how the study is conducted.

Although the Cronbach’s alpha of over 0.7 is considered desirable, values higher than 0.5 are acceptable, according to George and Mallery (2003) [74]. The whole questionnaire had a desirable Cronbach’s alpha with acceptable internal consistency based on the results obtained. Although the subscale’s values ​​obtained are not satisfactory, the results of Cronbach’s alpha should not be interpreted without considering the number of questions. One reason for low Cronbach’s alpha may be the low number of items in each dimension [75, 76].

The current study has several strengths and weaknesses. Some strengths of this study are as follows: a random sampling of the community-dwelling older adults, while different districts of the city in terms of development were considered in order to reach a representative sample; applying various methods of validity and reliability, such as the face, content, and construct validity, examining internal consistency and absolute reliability; designing a questionnaire with minimum items and maximum dimensions. The following can be mentioned as the weaknesses of the current study: due to the type of items and the range of different answers, performing exploratory factor analysis was impossible; the sample studied here was only the community-dwelling older adults who were not affected by cognitive disorders. Consequently, another psychometric assessment is necessary to enable the use of this questionnaire in other groups.

## Conclusion

Considering dimensions of health behaviors, which are of particular importance among the older adults, the questionnaire designed in the present study has created simple, short items to increase the answering pace and minimize the respondents’ burden. The results of this study revealed that the Geriatrics Health Behavior Questionnaire had good validity and reliability for use among Iranian community-dwelling older adults.

## Supplementary Information

**Additional file 1.** Appendix 1: Geriatrics Health Behavior Questionnaire (GHBQ) and Scoring method.

## Data Availability

All data supporting this study’s findings are not publicly available due to participants’ confidentiality; however, they are available from the corresponding author on reasonable request.

## Abbreviations

GHBQ: Geriatrics Health Behavior Questionnaire
CVR: Content validity ratio
K*: Modified Kappa statistic
CVI: Content validity index
I-CVI: Item content validity index
S-CVI/Ave: Scale content validity index
ICC: Intra-class correlation coefficient
CI: Confidence interval
SEM: Standard error measurement
MDC_95_: Minimal detectable change
SF8™: 8-item health survey
PCS: Physical component summary
MCS: Mental component summary
HPLP-II: Health-Promoting Lifestyle Profile II
MMAS: Morisky Medication Adherence Scale
USPSTF: United states preventive services task force
PSA: Prostate-specific antigen

## References

[1] Saghafi-Asl M, Vaghef-Mehrabany E (2017). Comprehensive comparison of malnutrition and its associated factors between nursing home and community dwelling elderly: a case-control study from northwestern Iran. Clin Nutr ESPEN.

[2] Hwang JE (2012). Development and validation of a 15-item lifestyle screening for community-dwelling older adults. Am J Occup Ther.

[3] Padula CA (1997). Development of the health-promotion activities of older adults measure. Public Health Nurs.

[4] Kulbok PA, Cox CL (2002). Dimensions of adolescent health behavior. J Adolesc Health.

[5] Bertera E (2002). Development and validation of a short health behaviors index for use with low socioeconomic status (SES) older adults. J Clin Geropsychol.

[6] Homan KJ, Boyatzis CJ (2010). Religiosity, sense of meaning, and health behavior in older adults. Int J Psychol Relig.

[7] Malmgren JA, Koepsell TD, Martin DP, Diehr P, LaCroix AZ (1999). Mortality, health services use, and health behavior in a cohort of well older adults. J Am Geriatr Soc.

[8] Martinez-Gomez D, Guallar-Castillon P, Leon-Munoz LM, Lopez-Garcia E, Rodriguez-Artalejo F. Combined impact of traditional and non-traditional health behaviors on mortality: a national prospective cohort study in Spanish older adults. BMC Med. 2013;11 10.1186/1741-7015-11-47. 10.1186/1741-7015-11-47 PMC3621845 23433432

[9] Michael YL, Colditz GA, Coakley E, Kawachi I (1999). Health behaviors, social networks, and healthy aging: cross-sectional evidence from the nurses' health study. Qual Life Res.

[10] Nigg CR, Long CR (2012). A systematic review of single health behavior change interventions vs. multiple health behavior change interventions among older adults. Translational. Behav Med.

[11] Rowe JW, Kahn RL (1997). Successful aging. The Gerontologist.

[12] Lee Y, Back JH, Kim J, Kim SH, Na DL, Cheong HK (2009). Systematic review of health behavioral risks and cognitive health in older adults. Int Psychogeriatr.

[13] Ha JY, Park YH (2015). Health status and factors related to health behaviors of older adults using a senior center. Korean J Adult Nurs.

[14] Luo YN, Zhu DW, Nicholas S, He P (2018). Depressive symptoms, health behaviors and risk of diabetes in Chinese mid-aged and older adults. J Affect Disord.

[15] Heine C, Browning C, Cowlishaw S, Kendig H (2013). Trajectories of older adults' hearing difficulties: examining the influence of health behaviors and social activity over 10 years. Geriatr Gerontol Int.

[16] Hamer M, Bates CJ, Mishra GD (2011). Multiple health behaviors and mortality risk in older adults. J Am Geriatr Soc.

[17] Walker SN, Sechrist KR, Pender NJ. The health-promoting lifestyle profile: development and psychometric characteristics. Nurs Res. 1987; https://pubmed.ncbi.nlm.nih.gov/3644262/3644262

[18] Pinar R, Celik R, Bahcecik N (2009). Reliability and construct validity of the health-promoting lifestyle profile II in an adult Turkish population. Nurs Res.

[19] Teng H-L, Yen M, Fetzer S (2010). Health promotion lifestyle profile-II: Chinese version short form. J Adv Nurs.

[20] Kuan G, Kueh YC, Abdullah N, Tai ELM (2019). Psychometric properties of the health-promoting lifestyle profile II: cross-cultural validation of the Malay language version. BMC Public Health.

[21] Tanjani PT, Azadbakht M, Garmaroudi G, Sahaf R, Fekrizadeh Z. Validity and reliability of health promoting lifestyle profile II in the Iranian elderly. Int J Prev Med. 2016;7 10.4103/2008-7802.182731. 10.4103/2008-7802.182731 PMC4882969 27280010

[22] Pérez-Fortis A, Ulla Diez SM, Padilla J-L (2012). Psychometric properties of the Spanish version of the health-promoting lifestyle profile II. Res Nurs Health.

[23] Tajik M, Galvão HM, Siqueira CE (2010). Health survey instrument development through a community-based participatory research approach: health promoting lifestyle profile (HPLP-II) and Brazilian immigrants in greater Boston. J Immigr Minor Health.

[24] Choi Y, Lee HJ (2016). Do regular cholesterol screenings Lead to lower cholesterol levels and better health behaviors for all? Spotlight on middle-aged and older adults in the United States. J Aging Health..

[25] Fernandez DM, Larson JL, Zikmund-Fisher BJ (2016). Associations between health literacy and preventive health behaviors among older adults: findings from the health and retirement study. BMC Public Health.

[26] Rabinowitz YG, Mausbach BT, Atkinson PJ, Gallagher-Thompson D (2009). The relationship between religiosity and health behaviors in female caregivers of older adults with dementia. Aging Ment Health.

[27] Tucker JS, Klein DJ, Elliott MN (2004). Social control of health behaviors: a comparison of young, middle-aged, and older adults. J Gerontol Ser B Psychol Sci Soc Sci.

[28] Whitehead BR (2016). Health behaviors in older adults: considering age, affect, and attitudes. J Health Psychol.

[29] Morisky DE, Green LW, Levine DM (1986). Concurrent and predictive validity of a self-reported measure of medication adherence. Med Care.

[30] Searle SD, Mitnitski A, Gahbauer EA, Gill TM, Rockwood K (2008). A standard procedure for creating a frailty index. BMC Geriatr.

[31] Polit DF, Beck CT, Owen SV (2007). Is the CVI an acceptable indicator of content validity? Appraisal and recommendations. Res Nurs Health..

[32] Polit DF, Beck CT (2006). The content validity index: are you sure you know what's being reported? Critique and recommendations. Res Nurs Health..

[33] Ayre C, Scally AJ (2014). Critical values for Lawshe's content validity ratio: revisiting the original methods of calculation. Meas Eval Couns Dev.

[34] Sadeghi R, Zanjari N (2017). The inequality of development in the 22 districts of Tehran Metropolis. Soc Welf.

[35] Lubkowska W, Krzepota J (2019). Quality of life and health behaviours of patients with low back pain. Phys Act Rev.

[36] Hong M, Shin H, De Gagne JC. Social networks, health-promoting behaviors, and health-related quality of life in older adults with and without arthritis. PLoS One. 2019;14(7) 10.1371/journal.pone.0220180. 10.1371/journal.pone.0220180 PMC6655701 31339940

[37] Kang H-W, Park M, Wallace JP (2018). The impact of perceived social support, loneliness, and physical activity on quality of life in south Korean older adults. J Sport Health Sci.

[38] McAuley E, Konopack JF, Motl RW, Morris KS, Doerksen SE, Rosengren KR (2006). Physical activity and quality of life in older adults: influence of health status and self-efficacy. Ann Behav Med.

[39] Hawkes AL, Pakenham KI, Chambers SK, Patrao TA, Courneya KS (2014). Effects of a multiple health behavior change intervention for colorectal Cancer survivors on psychosocial outcomes and quality of life: a randomized controlled trial. Ann Behav Med.

[40] Rejeski WJ, Mihalko SL (2001). Physical activity and quality of life in older adults. J Gerontol Ser A Biol Med Sci.

[41] Yiengprugsawan V, Kelly M, Tawatsupa B, Michalos AC (2014). SF-8TM health survey. Encyclopedia of quality of life and well-being research.

[42] Lefante JJ, Harmon GN, Ashby KM, Barnard D, Webber LS (2005). Use of the SF-8 to assess health-related quality of life for a chronically ill, low-income population participating in the Central Louisiana medication access program (CMAP). Qual Life Res.

[43] Namjoo S, Mirzaei M, Foroughan M, Ghaedamini HG (2021). Psychometric properties of the short Form-8 health survey (SF-8) among diabetes and non-diabetes Iranian older people. Health Promot Perspect.

[44] Cowell AJ (2006). The relationship between education and health behavior: some empirical evidence. Health Econ.

[45] Kenkel DS (1991). Health behavior, health knowledge, and schooling. J Polit Econ.

[46] Nunnally JC (1994). Psychometric theory 3E: Tata McGraw-hill education.

[47] Eisinga R, Grotenhuis M, Pelzer B (2013). The reliability of a two-item scale: Pearson, Cronbach, or spearman-Brown?. Int J Public Health.

[48] Mayers A (2013). Introduction to statistics and SPSS in psychology: Pearson Higher Ed.

[49] McDonald RP (2013). Test theory: a unified treatment: psychology press.

[50] Kirk J, Miller ML, Miller ML (1986). Reliability and validity in qualitative research: Sage.

[51] Cohen J (1988). Statistical power analysis for the behavioral sciences.

[52] Shoukri MM (2010). Measures of interobserver agreement and reliability.

[53] Cohen J (1960). A coefficient of agreement for nominal scales. Educ Psychol Meas.

[54] Landis JR, Koch GG. The measurement of observer agreement for categorical data. Biometrics. 1977:159–74. 10.2307/2529310. 843571

[55] Schougaard LMV, de Thurah A, Bech P, Hjollund NH, Christiansen DH (2018). Test-retest reliability and measurement error of the Danish WHO-5 well-being index in outpatients with epilepsy. Health Qual Life Outcomes.

[56] Nikpour M, Tirgar A, Ebadi A, Ghaffari F, Firouzbakht M, Hajiahmadi M (2018). Development and psychometric evaluation of a women shift workersâ€™ reproductive health questionnaire: study protocol for a sequential exploratory mixed-method study. Reprod Health.

[57] Troncoso C, Petermann-Rocha F, Brown R, Leiva AM, Martinez MA, Diaz-Martinez X (2018). Patterns of healthy lifestyle behaviours in older adults: findings from the Chilean National Health Survey 2009–2010. Exp Gerontol.

[58] Luo MY, Ding D, Bauman A, Negin J, Phongsavan P. Social engagement pattern, health behaviors and subjective well-being of older adults: an international perspective using WHO-SAGE survey data. Bmc. Public Health. 2020;20(1) 10.1186/s12889-019-7841-7. 10.1186/s12889-019-7841-7 PMC6979381 31973695

[59] Peltzer K, Pengpid S, Mohan K (2014). Prevalence of health behaviors and their associated factors among a sample of university students in India. Int J Adolesc Med Health.

[60] Luten KA, Dijkstra A, Reijneveld SA, De Winter AF (2016). Moderators of physical activity and healthy eating in an integrated community-based intervention for older adults. Eur J Pub Health.

[61] Yap AF, Thirumoorthy T, Kwan YH (2016). Systematic review of the barriers affecting medication adherence in older adults. Geriatr Gerontol Int.

[62] Paine P, Pasquali L, Ribeiro CAB (1998). Cognitive and value correlates of health-related behavior in Brazilian middle-class women. J Appl Soc Psychol.

[63] Xiang XL (2015). Chronic disease diagnosis as a teachable moment for health behavior changes among middle-aged and older adults. J Aging Health.

[64] Kitzman D, Haykowsky M (2017). Hazzard’s Geriatric Medicine and Gerontology.

[65] Neikrug AB, Ancoli-Israel S (2010). Sleep disorders in the older adult–a mini-review. Gerontology..

[66] Min Y, Slattum PW (2018). Poor sleep and risk of falls in community-dwelling older adults: a systematic review. J Appl Gerontol.

[67] Chaput J-P, Dutil C, Sampasa-Kanyinga H (2018). Sleeping hours: what is the ideal number and how does age impact this?. Nat Sci Sleep.

[68] Department of Health (2011). Physical activity, Health Improvement and Protection. Department of Health UK.

[69] World Health Organization (2003). Diet, nutrition, and the prevention of chronic diseases: report of a joint WHO/FAO expert consultation: world health Organization.

[70] Martin-Maria N, Caballero FF, Moreno-Agostino D, Olaya B, Haro JM, Ayuso-Mateos JL (2018). Relationship between subjective well-being and healthy lifestyle behaviours in older adults: a longitudinal study. Aging Ment Health.

[71] Abdelmawgoud A, Brown CJ, Sui X, Fonarow GC, Kokkinos PF, Bittner V (2015). Relationship of physical activity and healthy eating with mortality and incident heart failure among community-dwelling older adults with normal body mass index. ESC Heart Failure.

[72] Quinn K. Methodological considerations in surveys of older adults: technology matters. Int J Emerg Technol Soc. 2010;8(2) https://www.academia.edu/download/30841892/Article_4_Quinn.pdf

[73] Pärna K, Pürjer M-L, Ringmets I, Tekkel M (2014). Educational differences in cigarette smoking among adult population in Estonia, 1990–2010: does the trend fit the model of tobacco epidemic?. BMC Public Health.

[74] Geroge D, Mallery P (2003). SPSS for windows step by step: a simple guide and reference.

[75] Tavakol M, Dennick R (2011). Making sense of Cronbach's alpha. Int J Med Educ.

[76] Gliem JA, Gliem RR (2003). Calculating, interpreting, and reporting Cronbach's alpha reliability coefficient for Likert-type scales.

