# Supplementary material for: Development and validation of the geriatrics health behavior questionnaire (GHBQ)
Source: BMC Public Health. 2022 Mar 17;22:526. doi: 10.1186/s12889-022-12927-1 (PMC8932145; doi:10.1186/s12889-022-12927-1)
Supplement: Supplementary file 1 — Additional file 1. Appendix 1: Geriatrics Health Behavior Questionnaire (GHBQ) and Scoring method. [file 12889_2022_12927_MOESM1_ESM.docx]

**Appendix1: Geriatrics Health Behavior Questionnaire (GHBQ) and Scoring method**

**Authors:** Maryam Bakhshandeh Bavarsad, Mahshid Foroughan, Nasibeh Zanjari, Gholamreza Ghaedamini Harouni, Zahra Jorjoran Shushtari

| **Appendix1: Geriatrics Health Behavior Questionnaire (GHBQ)** | | | | | |
| --- | --- | --- | --- | --- | --- |
| **Subscales** | **Stem** | **Number of items** | **How to calculate** | **Scoring** | **Total score range** |
| Physical activity | 1a. On average how many times per week do you exercise?(consider activities that make breath faster and feel your body warm such as jogging, gardening, swimming, mountaineering, ping pong, and….) | 1 item | Item1a*Item1b | 0 min/week=0 | 0-1 |
|  |  |  |  | <150min/week=0.5 |  |
|  | 1b. How many minutes do you exercise each time? |  |  | ≥150min/week=1 |  |
| Nutrition Status | 2. Over the past month, how many serving of fruits have you eaten each day? (a serving means an apple or an orange, a slice of water melon, 2-4 plums or apricot, and 15 cherries) | 2 items | Sum of 2 items | 2 and less=0 | 0-2 |
|  | 3.Over the past month, how many serving of vegetables have you eaten each day?(a serving means a glass of raw vegetables or half a glass of cooked vegetables) |  |  | 3-4=0.5 |  |
|  |  |  |  | more than 5 times=1 |  |
| Medication adherence | 4. Have you ever forgotten to take your medicine? | 4items | Sum of 4 items | yes=0 | 0-4 |
|  | 5. Have you ever been careless to take your medicine? |  |  |  |  |
|  | 6. Have you ever stopped taking your medicine because you feel better? |  |  | no=1 |  |
|  | 7. Have you ever stopped taking your medicine because you feel worse? |  |  |  |  |
| Stress management | 8. When I am stressful, I often try to do something such as listening to music, talking to someone, gardening or… | 4items | Sum of 4 items | never=0 | 0-4 |
|  | 9. When I'm not able to change the situation, I will accept it. |  |  | occasionally=0.25 |  |
|  | 10.I think about good things while going to sleep |  |  | frequently=0.75 |  |
|  | 11. I know what increases my stress |  |  | always=1 |  |
| Smoking and alcohol consumption | 12. Which option shows your smoking status? | 2items | Sum of 2 items | I smoke=0 | 0-2 |
|  |  |  |  | I used to smoke=0.5 |  |
|  |  |  |  | I have never smoked=1 |  |
|  | 13. Which option shows your drinking behavior? |  |  | I drink usually=0 |  |
|  |  |  |  | I drink occasionally=0.5 |  |
|  |  |  |  | I never drink=1 |  |
| Sleep quality | 14. On average how many hours do you sleep in 24 hours? | 2items | Sum of 2 items | less than 7 or more than 9 hours per day=0 | 0-2 |
|  |  |  |  | 7-9 hours/day=1 |  |
|  | 15. Overall, how would you rate your sleep quality? |  |  | very bad=0 |  |
|  |  |  |  | bad=0.25 |  |
|  |  |  |  | moderate=0.5 |  |
|  |  |  |  | good=0.75 |  |
|  |  |  |  | very good=1 |  |
| Check Ups | 16. Have you met a dentist for regular check-ups in the last year? | 2items | Sum of 2 items | no=0 | 0-2 |
|  | 17. Have you had medical check Ups in the last year? |  |  | yes=1 |  |
| Total questionnaire |  | 17 items | Sum of subscales scores |  | 0-17 |
